# Missed Breast Cancers on MRI in High-Risk Patients: A Retrospective Case–Control Study

**DOI:** 10.3390/tomography8010027

**Published:** 2022-02-02

**Authors:** Julie Bilocq-Lacoste, Romuald Ferre, Grey Kuling, Anne L. Martel, Pascal N. Tyrrell, Siying Li, Guan Wang, Belinda Curpen

**Affiliations:** 1 Department of Diagnostic Radiology, University of Sherbrooke, Toronto, ON M4N 3M5, Canada; julie.bilocq-lacoste@usherbrooke.ca; 2 Radiology Department, University of Toronto, La Sarre Hospital, La Sarre, QC J9Z 2Y9, Canada; 3 Department of Medical Biophysics, University of Toronto, Toronto, ON M4N 3M5, Canada; grey.kuling@sri.utoronto.ca (G.K.); a.martel@utoronto.ca (A.L.M.); 4 Department of Statistical Sciences, Institute of Medical Science, University of Toronto, Toronto, ON M5T 1W7, Canada; pascal.tyrrell@utoronto.ca; 5 Department of Medical Imaging, University of Toronto, Toronto, ON M5T 1W7, Canada; siying.li@mail.utoronto.ca (S.L.); lilian.wang@mail.utoronto.ca (G.W.); 6 Department of Medical Imaging, Sunnybrook Health Sciences Centre, University of Toronto, Toronto, ON M4N 3M5, Canada; belinda.curpen@sunnybrook.ca

**Keywords:** breast MRI, high-risk screening, breast cancer, breast lesion detectability

## Abstract

*Purpose*: To determine if MRI features and molecular subtype influence the detectability of breast cancers on MRI in high-risk patients. *Methods and Materials*: Breast cancers in a high-risk population of 104 patients were diagnosed following MRI describing a BI-RADS 4–5 lesion. MRI characteristics at the time of diagnosis were compared with previous MRI, where a BI-RADS 1–2–3 lesion was described. *Results*: There were 77 false-negative MRIs. A total of 51 cancers were overlooked and 26 were misinterpreted. There was no association found between MRI characteristics, the receptor type and the frequency of missed cancers. The main factors for misinterpreted lesions were multiple breast lesions, prior biopsy/surgery and long-term stability. Lesions were mostly overlooked because of their small size and high background parenchymal enhancement. Among missed lesions, 50% of those with plateau kinetics on initial MRI changed for washout kinetics, and 65% of initially progressively enhancing lesions then showed plateau or washout kinetics. There were more basal-like tumours in BRCA1 carriers (50%) than in non-carriers (13%), *p* = 0.0001, OR = 6.714, 95% CI = [2.058–21.910]. The proportion of missed cancers was lower in BRCA carriers (59%) versus non-carriers (79%), *p* < 0.05, OR = 2.621, 95% CI = [1.02–6.74]. *Conclusions*: MRI characteristics or molecular subtype do not influence breast cancer detectability. Lesions in a post-surgical breast should be assessed with caution. Long-term stability does not rule out malignancy and multimodality evaluation is of added value. Lowering the biopsy threshold for lesions with an interval change in kinetics for a type 2 or 3 curve should be considered. There was a higher rate of interval cancers in BRCA 1 patients attributed to lesions more aggressive in nature.

## 1. Introduction

Multiparametric breast MRI is a highly sensitive modality used as part of the screening protocol for high-risk patients in Ontario’s High-Risk Ontario Breast Screening Program (HR OBSP) [1]. Although breast MRI has a high cancer detection rate [2,3], some challenging cases may contribute to missing breast cancers. This has already been described and published with mammograms. It has been shown that two-thirds of mammographically missed carcinomas are retrospectively visible [4]. Numerous causes affect MRI sensitivity, such as technically inadequate technical examination, smaller lesions and more extensive background enhancement [5]. Pages in [6] show that the main error in breast MRI interpretation is misinterpretation. In the same vein, the main missed breast cancer finding is a focus [7]. Kinetics pattern also contributes to false-negative cases [5,6,7,8,9].

Some references demonstrate that from a third to nearly half of breast cancers detected on MRI were visible in a prior screening study [6,7]. In contrast, patients with a genetic predisposition to breast cancer, such as carriers of the BRCA gene, are more likely to develop interval breast cancer [10].

We sought to investigate the missed breast cancers on MRI among high-risk patients, including the incident and prevalent ones. By influencing the detectability of breast cancers on MRI screening in a high-risk population, this study aims to determine which risk factors, features of breast lesions on MRI and type of tumour receptors may be associated with an increased risk of missed cancers on MRI.

## 2. Material and Methods

### 2.1. Inclusion and Exclusion Criteria

This retrospective case–control study investigates pathologically proved breast cancer cases documented with MRI. Research and ethics board approval was obtained.

Inclusion criteria required having a diagnosis of breast cancer within three months of an MRI describing a suspicious breast lesion (BI-RADS 4) or highly suspicious lesion (BI-RADS 5), with a prior MRI that was either normal (BI-RADS 1), benign (BI-RADS 2) or probably benign (BI-RADS 3).

Exclusion criteria included MRI biopsies corresponding to a premalignant condition (lobular carcinoma in situ), no MRI correlates for a mammographically visible finding and prior MR imaging archived outside of our PACS system.

Interval cancers were defined as lesions that were not visible on previous MRIs, while missed cancers were defined as those lesions which were visible on a retrospective review of the images.

Description of the lesions followed BI-RADS lexicon criteria. MR A was defined as the characteristics at the previous MRI. MR B was defined as the lesion’s MRI characteristics at the time of the diagnosis. Definition of MR A was the most recent MRI called normal, benign or probably benign (BI-RADS 1,2 or 3). MR B was defined as the MRI where the lesions were reported as suspicious/BI-RADS 4 or 5 for the first time.

The MRIs were interpreted by five fellowship-trained breast imagers with experience ranging from 4 to 30 years. One of the breast imagers was not fellowship trained and had more than ten years of experience. A women’s imaging fellow radiologist reviewed all the cases and discussed them with her supervisor. Other imaging studies, such as contemporary and past mammograms, were also reviewed on a case-by-case basis if this was relevant to data interpretation.

### 2.2. MRI Technique

Multiparametric contrast-enhanced MRIs were reviewed. Imaging was performed using a 1.5 T magnet (Magnetom; Siemens, Munich, Germany) and a dedicated breast coil. Localizer and bilateral T2, T1 non-fat sat, T1 pre and four dynamic runs post-gadolinium contrast (Gadovist^®^ 1.0, Bayer, Leverkusen, Germany) images were obtained right after contrast administration and after 2, 4 and 6 min. In total, 0.1 mmol/kg of IV gadolinium contrast was administered as a bolus.

Post-processing imaging included subtraction, and MIP and 3D reformatted MIP images were obtained. For the T1 sequence, the following parameters were used (Voxel size: 0.5 mm × 0.5 mm × 3.0 mm, FoV read 200 mm, slice thickness 3.0 mm, TR 5.39 ms, TE 2.39 ms, Flip angle 15.0 deg). For the T2 sequence, the following parameters were used (Voxel size: 0.6 mm × 0.6 mm × 3.0 mm, FoV read 200 mm, slice thickness 3.0 mm TR 6300.0 ms TE 79.0 ms, Flipangle 150 degrees).

### 2.3. Data Analysis and Interpretation

**Figure 1:**
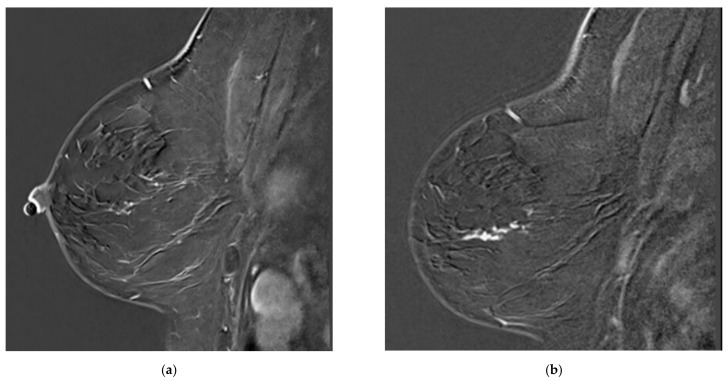
Examples of missed lesions. Overlooked linear non-mass enhancement: (**a**) MR A; (**b**) follow-up MRI (MR B) one year later showing more conspicuous non-mass enhancement, corresponding to DCIS.

**Figure 2:**
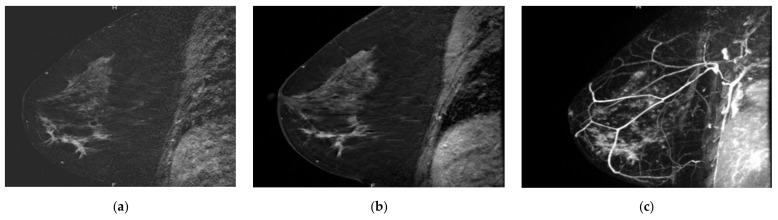
Overlooked segmental non-mass enhancement: (**a**) MR A; (**b**) MR B; (**c**) MR B MIP showing the enhancement standing out from BPE, corresponding to high-grade DCIS.

Breast lesions were first classified according to whether or not they were missed. The missed lesions were defined as visible on MR A (as a mass, non-mass enhancement, or focus standing out from background parenchymal enhancement) (Figure 1). The so-called missed lesions were subdivided into two categories, namely either overlooked, i.e., not described in the initial radiology report of MR A, or misinterpreted, either expressed as BI-RADS 2 or BI-RADS 3 on MR A, subsequently becoming BI-RADS 4 or BI-RADS 5 on MR B (Figure 1 and Figure 2). Non-missed lesions were defined as the absence of mass or non-mass enhancement on MR A. For each lesion, the MRI characteristics were documented according to the BI-RADS lexicon (T2 signal, size/volume, margins, distribution, enhancement and kinetics).

Background parenchymal enhancement was also documented, and the lesion was located at the previous lumpectomy or biopsy site. According to the BI-RADS lexicon, the breast lesions were manually measured in all three planes on the first acquired enhanced sequence to obtain a volume. A trained radiologist documented their MRI characteristics in a non-blinded fashion.

### 2.4. Statistical Analysis

The software SAS studio (SAS, Cary, NC, USA) was used for statistical analysis. The chi-square test and Fisher’s exact test for small samples were used to show if there was an association between the different imaging characteristics and whether a lesion was missed or not. The relationship between variables was then confirmed with a logistic regression model and the adjusted odds ratio and the corresponding 95% confidence interval were calculated. A one-way ANOVA test was used for volume comparison. A *p*-value of 0.05 was used to determine the threshold for statistically significant results.

## 3. Results

### 3.1. Patients

**Figure 3:**
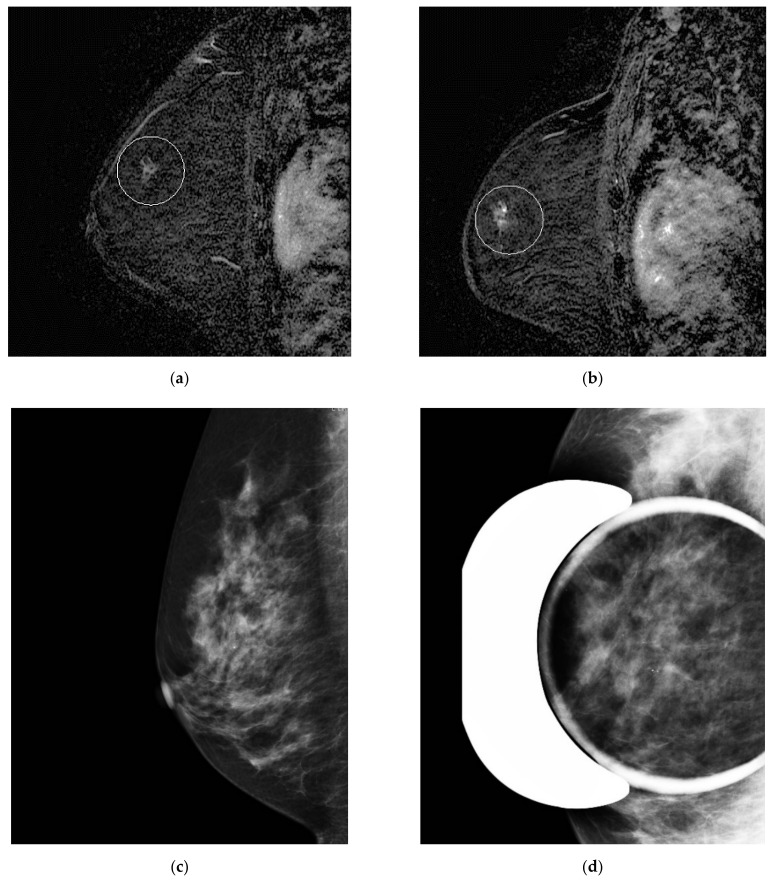
Misinterpreted stable non-mass enhancement between (**a**) MR A and (**b**) MR B. Calcifications were visualized in the same location and increasing in number on (**c**) the concomitant mammogram and (**d**) magnification views.

Our database included 22,000 breast MRIs from 7095 patients, carried out between 2005 and 2020. Of the 3000 malignancies investigated with MRI, 132 fulfilled our inclusion criteria, elaborated below. After excluding 28 patients, a total of 104 patients were reviewed. We consequently obtained 104 pathologically proved cancers for our analysis (Table 1). The reasons for excluding 28 patients were mostly because of inaccessible imaging data. In a few cases, patients initially were included via our research algorithm, but upon review turned out not to meet the criteria to be in the OBSP high-risk program: the biopsy results did not come back as malignant (for example in the case of in situ lobular carcinoma) or the cancerous lesion was only visible on mammogram as calcifications and not on MRI (Figure 3).

Indications for MRI were mostly screening (84 cases), follow-up of a BI-RADS 3 lesion (12 cases), cancer staging (6 cases) or the investigation of palpable clinical findings (2 cases) in high-risk patients.

The time span between two MRIs was between 6 and 26 months (mean 11.1 months). Patients’ ages ranged from 32 to 70 years old (mean age of 50.7 years old) and were all at high risk of breast cancer (lifetime risk equal to or greater than 25%, including women carrying the BRCA gene mutation and other high-risk syndromes). Among risk factors, 27 and 26 patients were carriers of BRCA 1 and BRCA 2 genes, respectively. Two had a previous history of chest radiation for lymphoma and one had Cowden’s syndrome.

### 3.2. Pathology

The breast cancers were of the invasive ductal carcinoma type in 55 cases, invasive lobular in 6 patients and DCIS in 38 cases. The remaining five cases were either mucinous, papillary or tubular. Among the invasive cancers, 48 were of the luminal type, 5 of the HER 2+ type and 19 of the basal-like sort. Available data from the pathology reports did not differentiate between luminal A and luminal B. There were four cases of HER 2+ equivocal cancers. Receptor status was not available for DCIS.

### 3.3. MRI Characteristics

**Figure 4:**
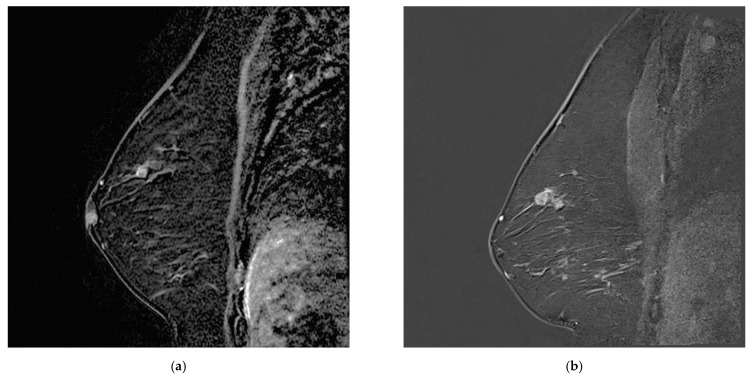
Increasing breast mass in a patient known for breast papillomatosis between (**a**) MR A, which was misinterpreted as a papilloma and (**b**) MR B, corresponding to papillary carcinoma.

**Figure 5:**
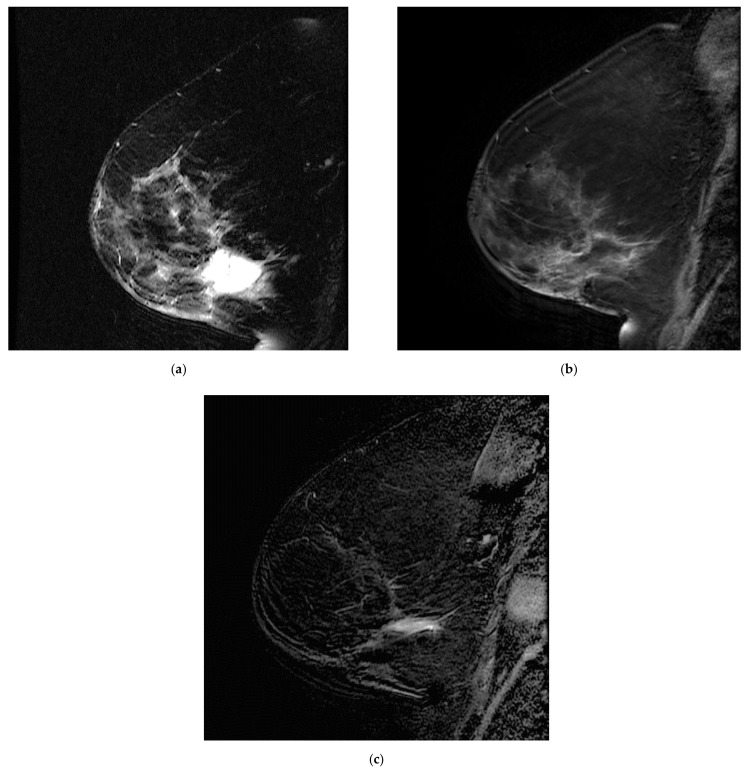
Postoperative seroma with surrounding non-mass enhancement on MRA (**a**) T2-weighted sequence and (**b**) T1 fat sat with gadolinium. The seroma resolved and linear enhancement is seen in the same location on (**c**) MR B. This was initially attributed to post-surgical changes and later confirmed to correspond to disease recurrence.

In total, 77 (74%) out of the 104 breast cancer cases included in our study were retrospectively visible on MR A and considered missed. In total, 51 were overlooked and 26 were misinterpreted (66% versus 34% of missed lesions, respectively). A total of 27 cases were non-missed cancers, i.e., classified as interval cancers (Figure 3, Figure 4 and Figure 5).

The size range of the breast lesions on imaging was from 0.2 cm to 2.4 cm for masses and 9.5 cm for non-mass enhancement. Most of the lesions measured between 0.5 cm and 1.5 cm.

### 3.4. Analysis

There is an association between BRCA1 gene carrying and missed cancers, more often overlooked in patients not carrying the BRCA1 gene (79%) compared with carriers (59%) (Table 1) OR 2.621, 95% CI [1.02, 6.74] *p* < 0.05.

There were more basal-like cancers in BRCA 1 carriers (50%) compared with non-carriers (13%) (Table 2). OR 6.714, 95% CI [2.058, 21.910] *p* < 0.05.

However, we did not take into account the very small groups, such as the chest radiation group, as statistically significant factors for missed cancers despite the below-threshold *p* value, given that the number of individuals in this group was too small.

There was no association between MRI characteristics (volume, distribution, margins, T2 signal, type and kinetics of enhancement) or background parenchymal enhancement and the frequency of missed cancers.

Some MRI characteristics differed according to the lesion’s receptors. HER 2+ cancers were more often in the form of non-mass enhancement than luminal and basal-like cancers (86% versus 28% and 12%, respectively), *p* < 0.05. Luminal and basal-like cancers were more often masses than HER 2+ cancers (72% and 88% versus 14%), *p* < 0.05. The odds ratio was 0.066 between HER 2+ and luminal (confidence interval 0.007–0.600) and 44.968 between HER2+ and basal-like, with a confidence interval of [3.408–593.864] (Table 3).

The breast lesions’ T2 signal remained unchanged for most lesions between MR A and MR B (72% and 81% for iso T2 signal and hyper T2 signal, respectively), *p* < 0.01. More than 50% of lesions also kept the same type of enhancement, *p* < 0.05.

Among the missed lesions, the majority (88%) kept the same kinetics on MR A and MR B in the ones with washout. Half of the lesions with plateau enhancement (50%) on MR A had washout enhancement on MR B. The majority (65%) of lesions with progressive enhancement on MR A had either plateau or washout enhancement on MR B (45% and 20%, respectively), Table 4.

There was no association between the type of receptors and the frequency of missed cancers. There was also no difference in the proportion of missed lesions between invasive cancers and DCIS. The ratio of overlooked versus misinterpreted lesions was also not significantly different depending on the receptor type.

MRI characteristics did not significantly differ between overlooked and misinterpreted lesions. In our retrospective review, qualitatively, of the six cancerous lesions at the site of previous biopsy or surgery missed, four (67%) were misinterpreted as a probably benign lesion for which a follow-up was recommended and one was overlooked. (Figure 4 and Figure 5). Other causes for misinterpretation included complex cases with multiple breast lesions (2 cases), longstanding stability in size (8 cases) and benign features such as smooth margins and hyper T2 signal (10 cases). Causes for overlooked lesions included increased background parenchymal enhancement (24 cases), faint enhancement (6 cases) and a small size of less than 5 mm (21 cases) (Table 5).

In the missed cancers category, in two cases, suspicious microcalcifications seen mammographically contributed to recognizing the non-mass enhancement as abnormal on MRI.

There was no significant difference in the volume of the lesions according to the type of receptors. A tendency was observed towards a more significant volume in HER 2+ cancers. However, this was non-significant.

## 4. Discussion

To our knowledge, our study comprises the highest number of false-negative breast MRIs, and this is the first study to compare the MRI characteristics of different molecular subtypes of breast cancer.

BRCA 1 and BRCA 2 gene mutations are the most common genetic predisposition to breast cancer [11]. Our results show that patients with the BRCA 1 gene mutation have a higher proportion of basal-like breast cancers. The cancers are less likely to be missed in those patients. The literature also reports a higher incidence of interval cancers in these patients, as the lesions rapidly evolve due to their histological type [8,9,12]. Triple-negative cancers are also more frequently associated with smooth margins [11,13], a confounding factor in identifying malignant breast lesions. Basal-like cancers alone were not a significant independent factor for missed lesions in our study. Given that missed cancers were not more frequent in a specific receptor type, regardless of the BRCA status, we cannot explain the higher rate of interval cancers in BRCA patients solely based on the higher proportion of basal-like tumours in this population. However, their aggressiveness and rapid evolution may not exclusively be related to the molecular subtype.

There was no relationship between MRI characteristics, receptors and risk factors for missed cancers. This may show that factors for missed lesions are independent of MRI characteristics or molecular subtypes. Qualitatively, lesions were most likely misinterpreted if the case was complex, such as the history of prior biopsy or surgery, or in a patient with multiple bilateral breast lesions or lesions that have remained stable for numerous years. Thus, this supports that lesion stability does not rule out malignancy, and any suspicious features should prompt further investigation, even if the lesion is stable in size. Therefore, a lower threshold for biopsy should be considered in those cases. Although non-significant, small size and high background parenchymal enhancement were more frequent in overlooked lesions. This is also in agreement with the literature [6,14]. Moreover, the correlation should be made with other modalities. In two cases, increasing calcifications on the mammogram associated with non-mass enhancement raised the suspicion for malignancy, even if the non-mass enhancement was stable.

Although no association between missed cancers, MRI characteristics and receptor subtypes was established, some of the findings’ reproducibility was validated in the literature. HER 2+ cancers were more often associated with non-mass enhancement, as previously noted in some studies [2,15], and a non-significant tendency towards more considerable size/volume. This may be because this type of cancer is presented more often in non-mass enhancement, which can be more spread out than masses, thus the more extensive measurements.

Among missed breast cancers, it was found that cancers with less suspicious kinetics (type 1 and 2 curves) on MR A often changed kinetics on MR B for a more suspicious, either type 2 or 3, kinetic curve (65% and 50%, respectively). Therefore, we consider that a change in kinetics, especially for more suspicious type 2 or 3 curves, may indicate cancer; thus, lowering the biopsy threshold should be considered in those cases. This was observed for all missed lesions, regardless of BRCA status, for our study. This agrees with Gilbert et al. [2], which was followed in BRCA patients, including small lesions of less than 5 mm, which more frequently had a type 1 curve. This supports the fact that kinetic curves should be assessed regardless of the size of the lesion, and the kinetic curves should be compared with the previous study, irrespective of if the previous kinetic curve was a type 1/non-suspicious curve.

Non-enhancing lesions and technically limited studies are also factors for missed lesions reported in the literature [6,7,16]. However, there were no cases of non-enhancing lesions in our cohort, and all studies were technically adequate.

The top category of missed cancers was overlooked lesions. This contradicts Pages et al.’s study [6], where more lesions were misinterpreted than overlooked. This can be partly explained by the situation in our centre, where there is good accessibility to MRI-guided biopsy. Therefore, the threshold for biopsy may be lower than in other centres. For instance, some lesions may be earlier characterized as BI-RADS 4 rather than as BI-RADS 3, thus diminishing the proportion of misinterpreted lesions. The studies were reviewed in a non-blinded fashion. MR A and MR B were reviewed together; this meant that even the very subtle lesions on MR A were categorized as “missed” and could have inflated the number of false-negative cases.

Finally, even though our study includes many cases of false-negative breast MRIs, the sample size remains relatively small, and a bigger sample size could have maximized the power of our study. A multi-institute study would also improve the generalizability of our results. Our study is a case–control study, not a double-blind study, which weakens some findings.

The radiologists’ experience was not taken into account as a factor that influenced missed cancer rate in this study. It may be an interesting factor to consider in future studies.

## 5. Conclusions

There is a high proportion (74%) of breast cancers visible on an initial MRI scan in a population of high-risk patients with serial MRIs. MRI characteristics or receptors do not influence the detectability of breast cancers. Lesions in a post-surgical breast or with moderate to high BPE should be assessed with caution. Long-term stability does not rule out malignancy, and multimodality evaluations are of added value. Lowering the threshold for biopsy for lesions with interval change in kinetics for type 2 or 3 curves should be considered. The kinetic curve of breast lesions should be compared with the previous study, regardless of the lesion size. The higher rate of interval cancers in BRCA 1 was not solely attributed to receptor type. However, this may be partly related to the higher proportion of basal-like cancers, which are more aggressive and more prevalent in those patients.

In future studies, evaluation of the missed cancers according to the radiologist’s experience could be performed. The creation of a data base containing examples of missed cases could also benefit trainees in order to familiarize them with the pitfalls of breast MRI and the causes of missed cancers encountered in the high-risk population.

## Figures and Tables

**Table 1:** Selection process of patients.

	*n*
Total breast MRIs	7095
Total malignancies detected	3000
Number of malignancies in high risk women with previous MRI	132
Number of patients excluded	28
Studies included in the analysis	104

**Table 2:** Breast cancer risk factors in missed and non-missed cancers. Number of patients according to breast cancer risk factors.

Risk Factor	Missed	Non-Missed	Total	*p* Value
	Number (Proportion of All Missed Lesions)	Number (Proportion of All Non-Missed Lesions)		
BRCA 1	16 (59%)	11 (41%)	27	<0.05
Non-BRCA 1	61 (79%)	16 (21%)	77	
BRCA 2	22 (85%)	4 (15%)	26	0.2
Non-BRCA 2	55 (71%)	23 (29%)	78	
Chest radiation	0	2 (100%)	2	0.07
No chest radiation	77 (75%)	25 (25%)	102	
Cowden	1 (100%)	0 (0%)	1	0.5
Non-Cowden	76 (74%)	27 (26%)	103	
Significant family history Without known syndrome	36 (80%)	9 (20%)	45	0.3
No significant family history	41 (69%)	18 (31%)	59	
Site of previous biopsy	5	0	5	0.2
Site of previous surgery	2	2	4	0.5

**Table 3:** Change enhancement kinetics between MR A and MR B in missed breast lesions.

Kinetics MR B	Kinetics MR A			
	Progressive	Plateau	Washout	*p*-Value
Progressive	8 (35%)	0	0	<0.05
Plateau	9 (45%)	14 (50%)	2 (12%)	
Washout	5 (20%)	14 (50%)	15 (88%)	
total	22	28	17	

**Table 4:** Breast cancer risk factors in different receptors.

Risk Factor	Luminal (Proportion of All Luminal Cancers)	HER 2+ (Proportion of All HER 2+ Cancers)	Basal-like (Proportion of All Basal-Like Cancers)	*p* Value
BRCA 1	5 (25%)	2 (10%)	10 (50%)	<0.05
Non-BRCA 1	41 (76%)	5 (9%)	7 (12%)	
BRCA 2	10 (66%)	0	4 (27%)	0.6
Non-BRCA 2	36 (61%)	7 (100%)	13 (22%)	
At the site of previous biopsy	0	1	2	0.06
At the site of previous surgery	1	1	0	0.5

**Table 5:** Identified causes for missed lesions.

Cause	Number of Cases
Small size	21
Increased background enhancement	24
Papillomatosis with multiple breast lesions	2
Long-standing stability in size	8
Benign features	10
Faint enhancement	6
Previous Biopsy or lumpectomy	6

## Data Availability

Data is available on the Biometrix data base at Sunnybrook Health Sciences Center.
